# The Effect of Extremely Low Frequency Pulsed Electromagnetic Field on *In Vitro* Fertilization Success Rate in N MRI Mice

**Published:** 2013-11-20

**Authors:** Leili Hafizi, Ameneh Sazgarnia, Nezhat Mousavifar, Mohammad Karimi, Saleh Ghorbani, Mohammad Reza Kazemi, Neda Emami Meibodi, Golkoo Hosseini, Hesam Mostafavi Toroghi

**Affiliations:** 1 Department of Obstetrics and Gynecology, Imam Reza Hospital, Mashhad University of Medical Sciences, Mashhad, Iran; 2 Department of Medical Physics, Mashhad University of Medical Sciences, Mashhad, Iran; 3 Department of Biomedical Engineering, Engineering Faculty, Islamic Azad University, Mashhad, Iran; 4 Biochemistry of Nutrition Research Center, Faculty of Medicine, Mashhad University of Medical Sciences, Mashhad, Iran

**Keywords:** *In Vitro* Fertilization, Electromagnetic Fields, Mice

## Abstract

**Objective:**

The effects of exposure to electromagnetic fields (EMF) on reproduction systems have been widely debated. In this study, we aimed to investigate whether low frequency EMF could ameliorate the *in vitro* fertilization success rate in Naval medical research institute (NMRI) Mice.

**Materials and Methods:**

In this randomized comparative animal study, ten NMRI mice were randomly divided into 2 equal groups (control and experimental). 10 IU of human chorionic gonadotropin (hCG) was injected intraperitoneally to both groups in order to stimulate ovulating, and ovums were then aspirated and kept in KSOM (modified version of sequential simplex optimization medium with a higher K^+^ concentration) culture medium. Metaphase II ovums were separated, and sperms obtained by "swim out" method were added to metaphase II ovums in the culture medium. The experimental group was exposed to 1.3 millitesla pulsed electromagnetic field at 4 kilohertz frequency for 5 hours. To assess the efficacy, we considered the identification of two-pronuclear zygote (2PN) under microscope as fertilizing criterion.

**Results:**

Total number of collected ovums in the control and experimental groups was 191 and 173, respectively, from which 58 (30.05%) and 52 (30.36%) ovums were collected from metaphase II, respectively. *In vitro* fertilization (IVF) success rate was 77% in extremely low frequency- pulsed electromagnetic field (ELFPEMF) for exposed group (experimental), whereas the rate was 68% for control group.

**Conclusion:**

Despite increased percentile of IVF success rate in exposed group, there was no statistically significant difference between 2 groups, but this hypothesis has still been stated as a question. Further studies with larger sample sizes and different EMF designs are suggested.

## Introduction

We are surrounded by many various magnetic, electric and electromagnetic fields (EMF). Natural electromagnetic fields are one of the factors maintaining the life in our planet. Pulsed electromagnetic fields (PEMF) stimulate many sub-cellular responses in living systems, but the range of action differs considerably depending on the properties of fields’ and types of cells and organisms. Plasma membrane, especially gap junctions and proteins connecting two adjuvant cells, are the most susceptible to such effects (1,2).

Although electromagnetic field has been subjected to various researches in recent years, it has been considered as a completely diverse and controversial subject, yet. Most of these researches have focused on machine life and effects of many electric instruments around us that mostly use50 Hertz EMF, which is as the target frequency evaluated by these studies. Some studies have insisted on the toxicological aspect of electromagnetic fields on various body systems and organs(3-5) including apoptosis (1,6,7), congenital anomalies (2), alteration in ion homeostasis (9), free radicals generation (9-11) and DNA damage (12,13), while some other researches have considered the therapeutic effects (14-17), especially on bone tissue(17,18). For example, extremely low frequency (ELF) is routinely applied in non-union factures (19), diabetic ulcer (20) and osteoarthritis treatment (21,22). In this category, the range of the frequency is almost wide, but mostly below 50 Hertz. Other positive effects of EMF include pain alleviation (acute and chronic) (23), inflammation reduction (24), nerve regeneration (25), improving blood flow (26), enhancement of delivery and effects of medications, angiogenesis (15,27), decreasing blood glucose and serum cortisol concentration (28) and so on.

There have been extensive researches about the effects of EMF on male reproductive system, but investigations about female reproductive system are scarce. About 15% of couples worldwide fail to give birth to a child (29). Assisted reproductive technology (ART) includes all methods involving laboratory manipulation of gametes (sperm or oocytes) and/or embryos for the purposes of reproduction. Although many diverse methods have been invented and employed, *in vitro* fertilization (IVF) is the most popular and preferable method in ART. Nowadays, IVF success rate is about 35% with a very short range of changes in different centers (30).

Regarding the potential *in vitro* effects of extremely low frequency- pulsed electromagnetic field (ELF-PEMF) on reproduction, growth and development, we aimed to investigate the effects of ELF-PEMF on IVF success rate in NMRI mice.

## Materials and Methods

The study was designed as a randomized comparative animal study.

### Animals

Ten female and 2 male Naval Medical Research Institute (NMRI) mice (20 to 25 g) were obtained from the Razi Institute (Mashhad, Iran) and housed under standard laboratory conditions. The maintenance and care of the mice complied with National Institutes of Health (NIH) guidelines for the humane use of laboratory animals. They were kept at constant room temperature (21 ± 2˚C) under a normal 12-hour light/12-hour dark cycle with free access to food and water. The study protocol was approved by the Ethics Committee of Research Council of Mashhad University of Medical Sciences. To identify possible effect of PEMF exposure on IVF success rate, female NMRI mice were randomly divided into 2 groups. Five mice were randomly selected for control group and five for experimental group.

During ovulation time, vaginal opening gets wet and its color changes into pink with high folding, so we used this sign to control the synchronization of their ovulation time.

### Induction of ovulation

To super ovulate the mice, we injected 10 IU of Pregnant Mare’s Serum Gonadotropin (PMSG; Sigma-Aldrich, St. Louis, USA) intraperitoneally, and 48 hours later, we injected 10IU human chorionic gonadotropin (hCG; Organon, Holland) intraperitoneally, as well. Thirteen hours after hCG injection, the mice were made unconscious and their abdomens were surgically opened under sterile conditions. The ovaries and fallopian tubes were dissected and transported into culture media. All ovums were aspirated under sterile conditions, transported into KSOM (Merck, Germany) culture media, and incubated at 37˚C temperature with 5% of CO_2_.

### Microscopic evaluation

In the next step, we put aspirated ovums in hyaluronidasesolution (Sigma-Aldrich, USA) for 1 minute to remove granulosa cell layer. Then, ovums, observed under stereo microscope (Olympus, Japan) for quality control, were counted consequently. Metaphase ΙΙ ovums were identified by their polar bodies and separated from other ovums.

### *In vitro* fertilization technique accomplishment

Metaphase ΙΙ ovums were kept in the culture media plate, separately, under aqua paraffin oil at 37˚C incubation for 1 hour (31). Meanwhile, to complete the IVF technique, male mice were killed, a section of their caudaepididymides was cut out, and placed into the culture media.

Afterwards, with punching and gentle pushing, sperms were squeezed out of epididymides and entered the culture media by swim out technique. Then, sperm suspension with the concentration of 5×10^4^ sperm/mL was prepared, mixed with metaphase ΙΙ ovums and transferred to fresh culture media.

In the final step, fertilization culture media of experimental group were placed under emitting of specified ELF-PEMF exposure system, and was then kept in the incubator for 5 hours.

Fertilization culture media of control group was kept in same incubation condition, without companionship of such designed system.

**Fig 1:**
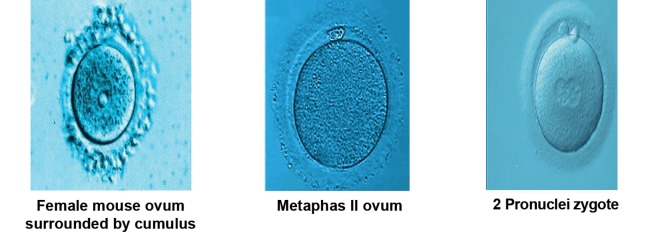
Mouse ovum in different stages of maturation and after inoculation

After 5 hours, the status of fertilization was investigated under microscope. The criterion of fertilization was to detect two pronuclei zygotes (2PN). Unfertilized ovums were counted separately (Fig 1).

### Pulsed electromagnetic field device set up

**Fig 2:**
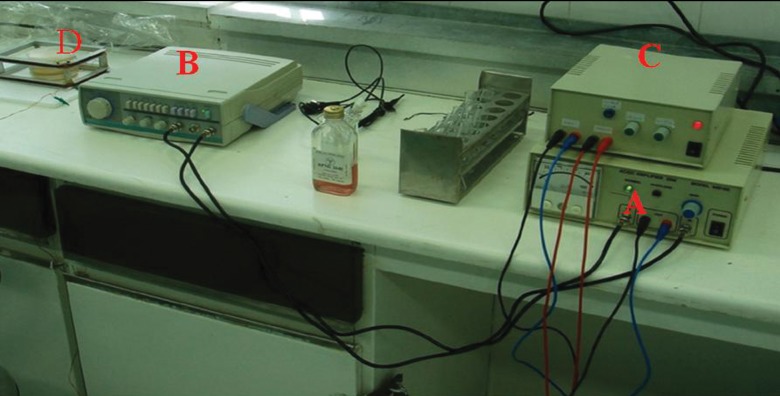
ELF- PEMF device settings. A. Wave generator, B. Amplifier 30 W, C. The designed circuit

The PEMF exposure device was previously stated in study by Hannay et al. (17). Briefly, two separate coils with dimensions 150×100 mm which was made up of 50 turns of 0.51 mm diameter acrylic coated copper wire were connected together in series and placed 20 mm apart. Each coil produced a resistance of 2.3 Ω. For inducing a parallel-aligned electric field, a PEMF pulse generator that produced a pulsed magnetic field perpendicular to the cell monolayer were wired to the coils. The PEMF signal contain 20 pulses of 5 milliseconds burst that was repeating at 15 Hertz. It was creating an asymmetrical "quasisquare wave" voltage trace during each burst at a frequency of ~4 kilohertz. Peak coil current duration lasted for 204 milliseconds, producing a maximum magnetic flux of 1.3 millitesla. The electromagnetic field strength induced inside the plate was identical thoroughly and independent to distance from the center (Fig 2).

### Statistical analysis

To compare the obtained results between experimental and control groups, Statistical Package for the Social Sciences (SPSS) software 16th release was carried out, whilechi-Square and Fisher’s exact test were applied to interpret the values. The p value under 0.05 was considered significant.

## Results

**Fig 3:**
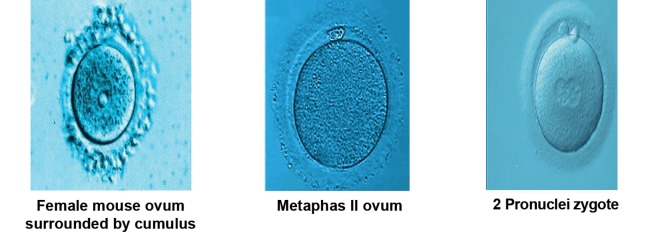
Frequencies of metaphase II ovums.

**Fig 4:**
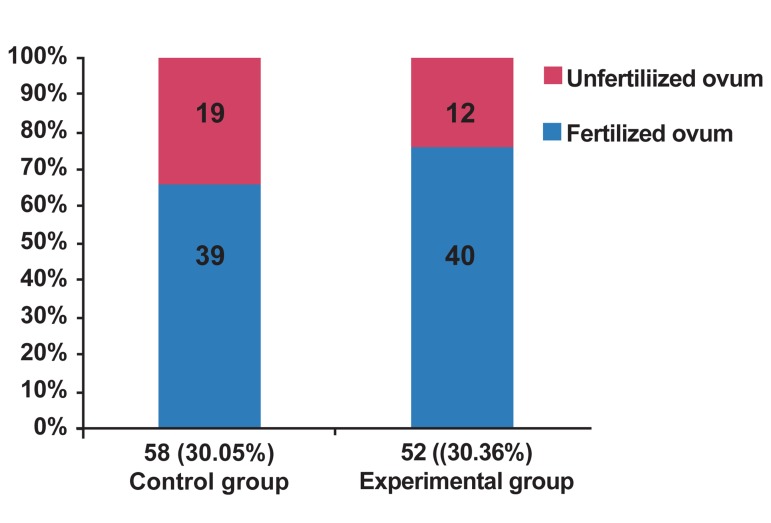
Comparison of percentage of fertilized and unfertilized ovums between two groups.

Total number of collected ovums in the control and experimental groups was 191 and 173, respectively, from which, 58 (30.05%) and 52 (30.36%) ovums were collected in metaphase II, respectively. Forming two pronuclei, which was considered as IVF success rate, was 77% in ELF-PEMF exposed group (experimental), while 68% in control group (Table 1,Fig 3,4).

**Table1:** Comparison of obtained results between two groups


Variable	Control group	Experimental group

**Total number of ovums**	191	173
**Ovums in metaphase II**	58 (30.05 %)	52 (30.36 %)
**Fertilized ovums**	39	40
**Unfertilized ovums**	19	12
**IVF success rate**	68%	77%


## Discussion

Due to undeniable negative social and economic consequences of infertility (32) and also failure in ART or other infertility therapies, it seems more attention should be devoted to improve the efficacy of methods.

The aim of this study was clarifying ELF-PEMF exposure effect on mice IVF success rate. The primary results showed an increase in forming couple pronuclei in ELF-PEMF exposed group, but that was a statistically insignificant increase. Consequently, we can say that exposing gametes to 50 Hertz pulsating electromagnetic field can influence the fertilization process.

Various hypotheses have been proposed for the mechanism of EMF action on cells. The most probable target for EMFs is the plasma membrane and transmembrane proteins rather than the cytoplasm. Gap junctions, specialized intercellular junctions, have been proposed as mediators of the EMF related cellular responses which change the cellular activity. One of the organelles responding to electromagnetic fields is microtubule, but this has not been proven yet (33,34).

Despite numerous studies about the effects of ELF-EMF on reproduction system, a paradigm has remained. Investigation of ELF-EMF on mammalian sperm showed both negative (3,4,35) and positive (14,16,36) effects. This controversy is also coherent with female fertility issues in which exposure to EMF was accompanied (37,38) or not accompanied (39,40) by significant adverse effects such as congenital anomaly. Ryan et al. (39) and Ohnishi et al. (40) have demonstrated that exposure to pure, linearly polarized 60 Hartz and power-frequency magnetic fields has no major effects on reproduction and development in mice. Tomás et al. (41) have also showed an increase in reproductive investment by breeding adults exposed to EMFs as compared to those in the adjacent reference area.

These findings have opened a new perspective and a growing interest for us to clarify whether ELF EMF ameliorate the IVF success rate in mice. It seems that no previous studies have been done about electromagnetic field as a promoting factor in mice IVF.

According to our previous unpublished data, we observed increased proliferation and differentiation of leukemic lymphoblasts after being exposed to ELF EMF emanating from a system same as in current study.

It is difficult to compare different studies due to many factors such as variety of frequency, intensity, timing and other magnetic properties which may interfere in results. But the structure of magnetic producer and circuit seems to be the most important factor.

## Conclusion

In this pilot animal study, we observed insignificantly improvement of IVF rate in NMRI mice. On the basis of vital effects of natural electromagnetic fields on cells and organisms, although our results were not significantly positive, we suggest investigating the effects of extremely low frequency PEMF (ELF-PEMF) on success rate of IVF on larger sample sizes of NMRI mice. We also strongly recommend more focusing on probable teratogens and other genetic disorders that may occur during ELF-PEMF exposure. One of our limitations in this study was small sample size.
